# Biomechanical Effect on Jack’s Test on Barefoot Position, Regular Socks, and Biomechanics Socks

**DOI:** 10.3390/life14020248

**Published:** 2024-02-11

**Authors:** Álvaro Gómez-Carrión, José Manuel Reguera-Medina, Manuel Coheña-Jiménez, Alfonso Martínez-Nova, Victor Manuel Jiménez-Cano, Rubén Sánchez-Gómez

**Affiliations:** 1 Nursing Department, Faculty of Nursing, Physiotherapy, and Podiatry, Universidad Complutense de Madrid, 28040 Madrid, Spain; alvgom25@ucm.es; 2 Podiatry Degree, Clinical Sanipie, Utrera, 41704 Sevilla, Spain; josemanuelremed22@gmail.com; 3 Podiatry Department, Faculty of Nursing, Physiotherapy, and Podiatry, Universidad de Sevilla, 41009 Sevilla, Spain; mcohena@us.es; 4 Nursing Department, Universidad de Extremadura, 10600 Plasencia, Spain; podoalf@unex.es (A.M.-N.); victormajc@unex.es (V.M.J.-C.)

**Keywords:** first metatarsophalangeal joint, proximal phalanx, Jack’s test, foot, sock, running

## Abstract

The proper dorsal flexion movement of the first metatarsophalangeal joint (MTPJ) is crucial for an accurate gait. Restricted movement can disrupt the windlass mechanism, and Jack’s test is a tool to assess such alterations. Although running socks are commonly used, their influence on the windlass mechanism remains unclear. Therefore, the aim of this study was to measure the resistance to passive dorsal flexion of the first metatarsophalangeal joint (MTPJ) under three different conditions: barefoot, wearing regular socks, and wearing biomechanical socks, using a digital force gauge. Methods: The research involved a sample size of 30 subjects (14 men and 16 women), and Jack’s test was conducted using a digital force gauge and a lever system. Three conditions were measured, barefoot, with a regular sock, and with the biomechanical socks. Results: Statistically significant differences were observed when using biomechanical socks with orthopedic corrections during Jack’s test, as measured with the digital force gauge (13.33 N ± 3.54, *p* < 0.001). Conclusions: The utilization of biomechanical socks with a kinetic wedge, reinforced mesh in the medial longitudinal arch, and padding in the heel area results in a reduction of the force required, measured in newtons, to perform dorsal flexion of the first metatarsophalangeal joint (MTPJ) during Jack’s test compared to being barefoot or wearing regular socks.

## 1. Introduction

The mechanism of windlass occurs in the foot when the hallux is extended. This mechanism involves the plantar fascia and leads to plantar flexion of the head of the first metatarsal, an increase in the height of the medial longitudinal arch (MLA) of the foot, and an inversion movement of the calcaneus. During the gait push-off phase, it generates an external rotation of the lower limb and supination of the subtalar joint, unaided by muscle action [1,2,3,4]. The primary joint involved in the windlass mechanism is the first metatarsophalangeal joint of the hallux. This joint has a range of motion of 30 to 50 degrees during physiological gait [5]. When measuring the range of motion of the first MTPJ with a goniometer and it falls below the physiological range, it is considered as hallux limitus. If there is a limitation of movement in the first metatarsophalangeal joint, it indicates hallux limitus, which is associated with an inefficient gait [6]. The limitation of extension in the lower limb during the push-off phase of running is linked to an inefficient gait, often associated with a restriction in dorsal flexion of the first metatarsophalangeal joint (MTPJ). Alterations in movement along the sagittal plane can trigger biomechanical changes that may impact the lower back, ankle, and foot [7,8,9,10,11].

A maneuver employed in the clinical community is the Jack’s test, conducted with the subject in a barefoot standing position. The examiner performs a passive dorsal flexion of the hallux phalanx, simultaneously increasing the height of the medial longitudinal arch of the foot. The test is considered physiological when, during passive dorsal flexion without a decrease in the range of motion, a minimal force is required to generate the increase in the height of the medial longitudinal arch of the foot [3,12]. This maneuver enables the clinician to examine the force exerted during passive dorsal flexion of the hallux and to understand the forces acting on the foot. Two devices have previously been used to quantify the resistance to passive dorsiflexion of the hallux: one used in Moisan’s research [12] and the other developed by Gómez’s research group [13]. The latter achieved reliable means of measurement.

For the past 50 years, running has been one of the most popular and accessible sports practiced worldwide. This sport has a low cost and can be pursued with minimal equipment, contributing to the significant increase in its number of participants in recent decades. Additionally, running promotes a healthy lifestyle within society [14,15]. This sports activity is associated with the prevention of cardiovascular diseases and the enhancement of longevity. Running can not only improve overall health but also contribute to the management and enhancement of well-being [16]. Apart from the health benefits, there are also pathologies associated with running. Running-related pathologies typically entail a slow recovery process, necessitating a prolonged reduction or elimination of sports activity. These pathologies manifest as specific lesions, predominantly occurring in the lower limb. The prevalence of such lesions is notably high in anatomical areas encompassing the hip, knee, ankle, and foot [15].

This prevalence is due to the fact that the foot is the structure in direct contact with the ground, and any deformity or alteration can give rise to proximal pathological forces. The MLA of the foot plays a crucial role in absorbing ground reactive forces and cushioning the impact with the ground [17]. The morphology of a diminished MLA is associated with an increased risk of injury during running and impedes proper foot function [18]. A decrease in MLA not only has consequences in pathomechanics, but the presence of a higher MLA also amplifies the ground reactive forces in the medial forefoot. Common pathologies linked to a decreased arch include medial tibial stress, patellofemoral syndrome, tibialis posterior dysfunction, and plantar fasciopathy [19,20,21,22].

The use of compressive running socks is widespread among marathon runners. These socks are primarily utilized to enhance blood circulation in the legs, minimize muscle oscillation, improve oxygen supply, and reduce fatigue during a run [23]. We know that their use is related to the improvement of ankle proprioception during running, which can enhance sports performance and biomechanics during the activity [24]. Recently, the first biomechanical socks (Podoks^®^) have been designed, featuring a kinetic wedge [25], a reinforcement mesh in the medial longitudinal arch (MLA), and padding in the heel area. The effect of this new sock design on the windlass mechanism and the Jack’s test is not yet known. Therefore, the aim of this study was to measure the resistance to passive dorsal flexion of the first metatarsophalangeal joint (MTPJ) under three different conditions: barefoot, wearing regular socks, and wearing biomechanical socks, using a digital force gauge. This study hypothesizes that the use of biomechanical socks reduces the force required during Jack’s test.

## 2. Material and Methods

This research project was submitted to the University of Plasencia, recognized as a public institution, to seek approval from the Ethics Committee (ID: 89//2023), specifically the Bioethics and Biosafety Committee of the University of Extremadura (CBBUEx). The research adhered to ethical and human criteria outlined in the Declaration of Helsinki. A dedicated informed consent form was developed for the study, and all participants signed it before participating. Stringent measures were implemented to ensure the anonymity and privacy of participants’ data, following Organic Law 15/99 of 13 December.

### 2.1. Participants

The sample size for this research was determined by the Statistical Unit of the Complutense University of Madrid. This study falls under the classification of Strengthening the Reporting of Observational Studies in Epidemiology (STROBE). The objective was to investigate potential differences during Jack’s test under three different conditions: barefoot, regular socks, and biomechanical socks. The sample size was calculated using G*power software (version 3.1.9.6, Kiel University, Kiel, Germany), based on previous data from similar research studies [13]. Based on statistical considerations, for a power level of 80%, a confidence interval (CI) of 95%, β (beta) set at 20%, and α (alpha) at 0.05, the study required the inclusion of 30 subjects [13]. To account for potential participant losses, a total of 45 subjects were chosen.

All subjects needed to meet the inclusion criteria: (1) Subjects of both sexes aged over 18 years and under 65 years [12]; (2) Range of motion of more than 30° of dorsal flexion of the first MTPJ [12,26]; (3) Neutral foot result (0 to +5) measured with the Foot Posture Index (FPI) [27,28]. There were only three exclusion criteria: (1) Present previous surgery on the foot; (2) Alteration of balance diagnosed; (3) Diagnosis of neurology alteration [12]. The subjects were recruited from the podiatry unit of the San Agustín Hospital (Seville), during April to May 2023.

### 2.2. Procedures

Subjects were assessed to determine their eligibility based on the study’s inclusion criteria. This assessment was carried out via face-to-face interviews conducted by a research team member. Participants were fully briefed about the study procedures, and upon their agreement to participate, they were required to read and sign an informed consent form. Subsequently, demographic data of each subject, including age, height, weight, and history of lower limb injuries, were collected. The study also involved measuring the Foot Posture Index (FPI), a widely recognized and valid tool used in numerous studies for classifying foot types. The FPI allows for the categorization of feet into three types: supinated, neutral, or pronated, and is known for its reliability, with an intraclass correlation coefficient ranging from 0.62 to 0.91. The Foot Posture Index (FPI) comprises six criteria for evaluation, which collectively provide a comprehensive assessment of foot posture. These criteria include: Palpation of the Talus Head: This involves assessing the position and prominence of the talus head. It gives an indication of the arch’s height and the foot’s overall alignment. Supra and Infra Malleolar Curvature: This criterion evaluates the curvature above (supra) and below (infra) the ankle malleoli. It helps to determine the tibial and lower leg alignment and its effect on foot posture. Prominence in the Talonavicular Joint: This assesses the degree of prominence or protrusion of the talonavicular joint, which can indicate arch height and alignment. Height of the Arch: Direct measurement of the arch height is crucial in determining whether the foot is flat, normal, or has a high arch. Position of the Calcaneus in the Frontal Plane: This looks at how the heel bone (calcaneus) is aligned in the plane that divides the body into front and back, helping to assess overall foot alignment. Position of the Forefoot in Relation to the Rearfoot: This assesses the alignment of the forefoot in relation to the rearfoot, indicating potential issues like overpronation or supination. For inclusion in this study, subjects were required to have an FPI score ranging from 0 to +5. This range likely represents a normal or neutral foot posture, excluding those with extreme pronation or supination, which could affect the study’s outcomes regarding the biomechanical effects of different situations on the foot during the Jack’s test [27,28,29]. The range of motion of the first metatarsophalangeal joint (MTJP) was assessed using a goniometer, with one arm positioned on the proximal phalanx and the other on the metatarsal. A range of motion exceeding 30° was necessary [26]. A mark was placed on the proximal phalanx of the first metatarsophalangeal joint (MTJP) using a dermatographic pencil, following the protocol outlined in Heng’s study [30].

**Figure 1:**
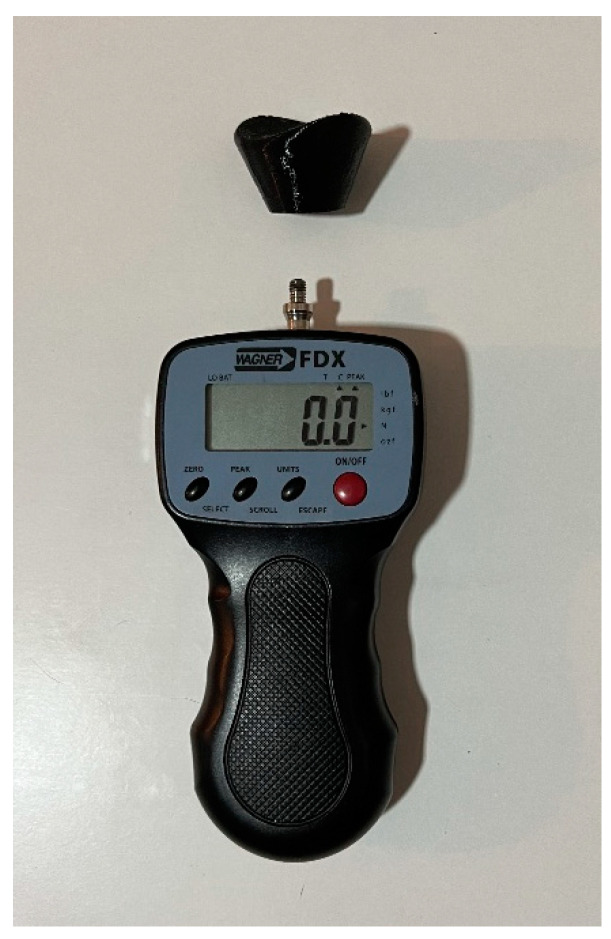
The digital force gauge with Hallux adapter (FPX^®^ 25, Wagner Instruments^®^, Greenwich, CT, USA).

**Figure 2:**
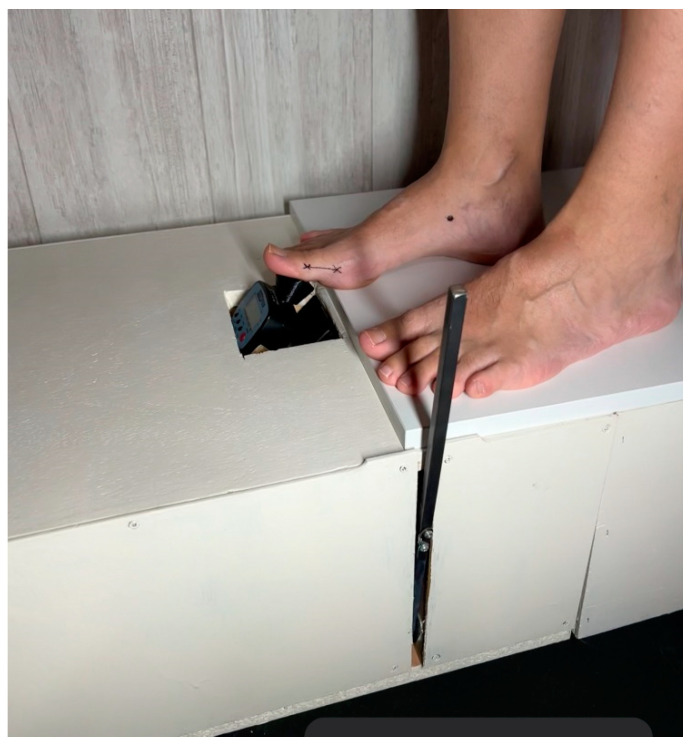
The subject in barefoot position for measuring Jack’s test with the digital force gauge.

**Figure 3:**
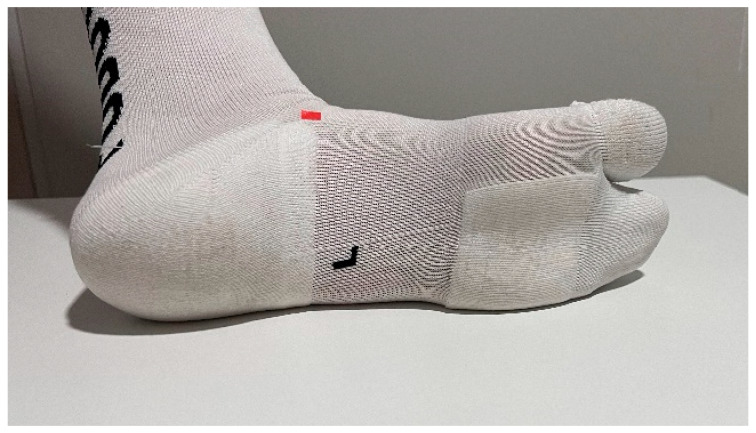
The subjects with biomechanics socks.

**Figure 4:**
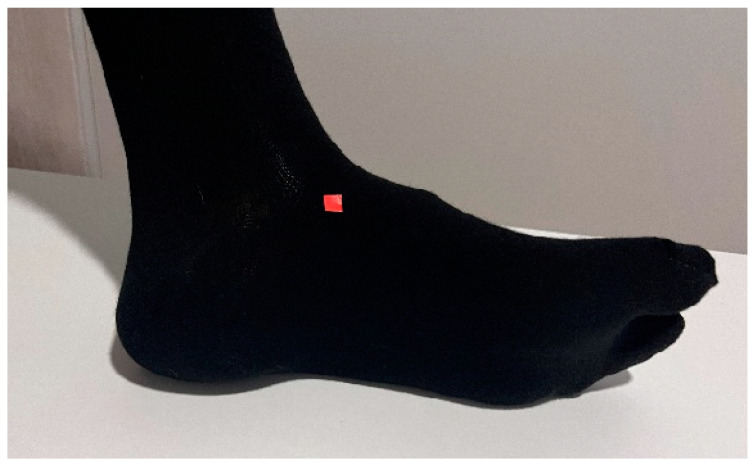
The subjects with regular socks.

The same protocol for measuring passive dorsal flexion of the hallux, as described in Gomez’s research, was followed [13]. The Jack’s test measurement was conducted using a digital force gauge (FPX^®^ 25, Wagner Instruments^®^, Greenwich, CT, USA) (Figure 1) [31,32,33]. The head of the digital force gauge was positioned at the mark on the proximal phalanx of the hallux to ensure a consistent lever arm. A 45° inclination relative to the proximal phalanx of the hallux was maintained [30]. The initial researcher employed a lever and pulley system to quantify the force required for conducting Jack’s test on the subject (Figure 2). The application speed of the lever was consistently maintained. Marks were made on the navicular bone in three different situations to identify the moment of height increase. The digital force gauge recorded the force in newtons when the windlass mechanism was activated [12]. There were two researchers conducting the measurements. Each condition was measured three times by each researcher, resulting in a total of six repetitions for each condition. The socks were randomly assigned to avoid any order effect, and a wait time of 10 s was implemented between each measurement. During the research, three different conditions were assessed: barefoot, wearing regular socks without any additional features or corrective elements (Figure 3), and wearing biomechanical socks (Figure 4). The participants were unaware of the type of sock they were wearing in each test. Measurements were conducted on the dominant foot, though socks were worn on both feet to prevent any changes in sensation.

### 2.3. Statistical Analysis

The Complutense University of Madrid’s computing center conducted the statistical analysis using SPSS Version 20.0 for Windows (IBM Corp, Armonk, NY, USA). The analysis aimed to determine if the sample followed a normal distribution. The Kolmogorov–Smirnov test indicated a non-normal distribution of the sample (*p* < 0.05). Given the sample’s non-normality, nonparametric tests were deemed appropriate.

The nonparametric Friedman test was utilized to analyze if there were significant differences across the three different conditions: barefoot, wearing regular socks, and wearing biomechanical socks. Additionally, the Wilcoxon test, another nonparametric test, was employed to ascertain if there were significant differences between being barefoot and wearing each type of sock.

## 3. Results

A total of 45 subjects were initially included in this research to account for the common loss of 20%. However, due to participant withdrawal, the study concluded with a total of 30 subjects. Fifteen subjects withdrew, and their data were not included in the analysis. The final sample comprised 30 subjects, evenly divided between men and women (14 men and 16 women), and the demographic data revealed a mean age of 44.87 ± 18.86 years, a mean weight of 66.40 ± 12.04 kg, a mean height of 165.73 ± 8.18 cm, and a mean Foot Posture Index (FPI) of 2 ± 1.39 (Table 1).

The reproducibility of the data obtained for measurements in barefoot position, with regular socks, and with biomechanical socks is presented using Intraclass Correlation Coefficients (ICCs), the Minimal Detectable Change (MDC), and the Standard Error of Measurement (SEM), as shown in Table 2. For the barefoot position, the ICC for intratester reliability ranged from 0.989 to 0.995. With regular socks, the ICC for intratester reliability ranged from 0.992 to 0.998, and for biomechanical socks, it ranged from 0.990 to 0.997. The SEM for the barefoot position was 0.262, for regular socks it was 0.345, and for biomechanical socks it was 0.269. The MDC results were 0.841 for the barefoot position, 0.995 for regular socks, and 0.745 for biomechanical socks. The ICC for intertester reliability was 0.983 for the barefoot position, 0.971 for regular socks, and 0.953 for biomechanical socks. According to the Landis and Koch Criteria for ICC, a result between 0.81 and 1 indicates nearly perfect agreement. These ICC results suggest almost perfect agreement in the sample data [34,35]. Across all measures, the mean ± SD for the 30 feet (two clinicians with three measurements each) was 20.21 ± 2.72 N for the barefoot position, 19.62 ± 5.18 N for regular socks, and 13.33 ± 3.54 N for biomechanical socks. The force measured in newtons during passive dorsal flexion of the phalanx of the first MTPJ was lower with the use of biomechanical socks compared to regular socks and the barefoot position (*p* < 0.001), as detailed in Table 3.

## 4. Discussion

The present study aimed to measure the force required in newtons to perform dorsal flexion of the first MTPJ under three different conditions. The results of this research indicate that when using biomechanics socks, the force needed for dorsal flexion of the first MTPJ is lower than with regular socks and in a barefoot position (*p* < 0.001). According to Dananberg’s studies, reducing tension in the plantar fascia enhances the integration of the windlass mechanism. By using a kinetic wedge, like the one incorporated in biomechanics socks, the ground reactive forces under the head of the first metatarsal are diminished. This reduction makes it easier to activate the windlass mechanism during gait. Such facilitation in activating the windlass mechanism leads to an increase in the height of the MLA, supination of the rearfoot, and external rotation, all occurring with less tensile force in the plantar fascia [12,14,28]. Our results align with those of this author, showing that the use of orthopedic corrections embedded in the sock reduces fascial tension during the Jack’s test. While our study showed a mean force of 58.3 ± 14.5 N during dorsal flexion of the first MTPJ, our results deviate from those reported by Moisan [12]. Our mean force was 19.62 ± 5.18 N for regular socks without correction. This discrepancy may be attributed to Moisan’s inclusion of subjects with FPI scores greater than 5 and higher body mass. Additionally, differences in devices used could contribute to varying lever arms, impacting the newtons generated across the two studies. However, it is worth noting that our study demonstrated excellent reliability, as reflected in the Intraclass Correlation Coefficient (ICC), which aligns with Moisan’s findings. This indicates that the data obtained from both studies demonstrate satisfactory performance for the measuring devices.

The results from Sánchez’s study [31] indicate an average force of 32.55 ± 1.27 N during dorsal flexion, utilizing the same algometer employed in our research. However, our findings diverged, revealing lower force values. One notable distinction lies in the application technique of the algometer to the proximal phalanx—our study employed a thrust movement, whereas Sánchez’s study used a traction movement. Another potential factor is the utilization of the Foot Posture Index (FPI) in our study for categorizing foot types, a practice not adopted in Sánchez’s study. Despite these differences, both investigations employed the same algometer, yielding similar results; Sánchez reported an Intraclass Correlation Coefficient (ICC) of 0.971, while our research achieved an ICC of 0.995. The research conducted by Heng (30) demonstrated that his new device could effectively measure the strength required to activate the windlass mechanism. He reported an Intraclass Correlation Coefficient (ICC) of 0.814, which is similar to the ‘excellent’ reliability found in our research. A key difference between the two studies is the position used during the maneuver: Heng’s research was conducted in a non-weight-bearing position, whereas our study was performed in a weight-bearing, barefoot position. In Armstrong’s study [36], improvements were observed with the application of compression socks during running. We posit that this compression enhances the stability of the medial longitudinal arch (MLA), thereby reducing the descent of the navicular and promoting a more efficient windlass mechanism. Our results align with this notion, as the biomechanics socks feature a compressive reinforcement within the MLA, controlling its descent. This design helps diminish tensile stress on the plantar fascia, ultimately contributing to the enhancement of the windlass mechanism. Another study aligning with these findings is Mota’s [37] systematic review. In it, Mota argues that, based on the studies reviewed, the use of running socks appears to enhance an individual’s sensory perception during movement. These findings are intriguing because running involves repetitive movements, and any restriction during the sagittal plane could disrupt the fluidity of these motions. The enhanced windlass mechanism, as suggested by our research, has the potential to alleviate such restrictions. Our results align with the hypothesis that the improved activation of this mechanism contributes to this positive outcome. Furthermore, the results from [38] indicate that using compression socks in sports can induce changes in the peripheral nervous system and biomechanical alterations in the hip and knee joints. These outcomes are consistent with our study, underscoring how the use of corrected socks can enhance foot functionality, particularly in strengthening dorsiflexion of the first MTPJ.

The strengths of this research are that the use of the socks improves the windlass mechanism, decreasing the force to activate it. This improvement can help the individual reduce the tensile stress on the plantar fascia during running. It could also delay race fatigue. The measures collected in this research have good reproducibility of intraclass correlation coefficients. The clinical application would be indicated in pathologies such as plantar fascia or hallux limitus where the patient presents alteration of the windlass mechanism, and the use of these new socks could provide improvements in the symptomatology. Also, pathologies where the pronator moments generate a delay in the activation of the windlass mechanism can benefit from the use of these socks with orthopedic corrections.

Future lines of research would be aimed at applying these new socks with use in subjects with foot pathology and assessing how their symptoms evolve. Electromyograms can also be used to find out how improvements in the windlass mechanism would have an impact on the muscles of the lower limb. Also, the use of different orthopedic pieces and thicknesses in socks could be used to evaluate how it affects the foot, to be able to measure how the force affects the windlass mechanism by changing the angulation of the proximal phalanx, and to see the tensile stress received.

### Limitations

This study has certain limitations. The use of a lever to control the displacement of the algometer and the speed at which it is executed introduce potential sources of error. The positioning of the algometer during measurement could induce skin discomfort in certain individuals. For patients with sweaty feet, it was essential to dry the skin to prevent blistering, and researchers were trained to handle this aspect appropriately. The potential instability associated with being on an elevated structure may also impact accurate data collection. Additionally, the discomfort associated with wearing specialized compressive running socks with orthopedic corrections could potentially influence data collection. The strengths of this research lie in the demonstrated enhancement of the windlass mechanism through the use of socks, resulting in a reduced force requirement for activation. This improvement holds potential benefits for individuals by minimizing tensile stress on the plantar fascia during running and possibly delaying race-related fatigue. The measurements collected in this study exhibit good reproducibility, as indicated by the intraclass correlation coefficients.

Clinically, the application of these findings could be valuable in conditions such as plantar fasciitis or hallux limitus, where patients exhibit alterations in the windlass mechanism. The use of these innovative socks, incorporating orthopedic corrections, may offer symptomatic improvements. Additionally, pathologies characterized by pronator moments causing delayed activation of the windlass mechanism could also benefit from these socks.

Future research avenues could explore the application of these socks in individuals with existing foot pathologies, tracking the evolution of their symptoms. Electromyograms could be employed to investigate the impact of improved windlass mechanism on lower limb muscles. Exploring variations in orthopedic components and sock thickness may provide insights into their effects on the foot. Assessing the influence of force on the windlass mechanism by altering the angulation of the proximal phalanx could further illuminate the dynamics involved and the resultant tensile stress.

## 5. Conclusions

The utilization of biomechanical socks with a kinetic wedge, reinforced mesh in the medial longitudinal arch, and padding in the heel area results in a reduction of the force required, measured in newtons, to perform dorsal flexion of the first metatarsophalangeal joint (MTPJ) during Jack’s test compared to being barefoot or wearing regular socks.

## Figures and Tables

**Table 1:** Summary anthropometric measurements.

	N	Mean	SD	Minimum	Maximum
Age (Years)	30	44.87	18.86	18	64
Weight (kg)	30	66.40	12.04	46	85
Heigth (cm)	30	165.73	8.18	155	181
FPI (Scores)	30	2.00	1.39	0	5

Abbreviations: **SD** = Standard Deviation; **FPI** = Foot Posture Index.

**Table 2:** The table shows the reliability of the data with the ICCs, SEM, and MDC in newtons for the three different situations.

	Barefoot					Regular Socks					Biomechanics Socks				
Variables	SD	ICC Intratester	ICC Intertester	SEM	MDC	SD	ICC Intratester	ICC Intertester	SEM	MDC	SD	ICC Intratester	ICC Intertester	SEM	MDC
		(95% CI)	(95% CI)				(95% CI)	(95% CI)				(95% CI)	(95% CI)		
Newtons (N)	4.61	0.991	0.983	0.262	0.841	5.18	0.995	0.971	0.345	0.995	3.54	0.995	0.953	0.269	0.745
		(0.989–0.995)					(0.992–0.998)					(0.990–0.997)			

Abbreviations: **SD** = Standard Deviation; **ICC** = Intraclass Correlation Coefficient; **SEM** = Standard Error of Measurement; **MDC** = Minimal Detectable Change; **CI** = Confidence Interval.

**Table 3:** Newtons force results with the use of each situation.

Variables	Barefoot Mean (Newtons) ± SD (95% CI)	Regular Socks Mean (Newtons) ± SD (95% CI)	Biomechanics Socks Mean (Newtons) ± SD (95% CI)	*p* Value Regular Socks vs. Barefoot Mean (Newtons) ± SD (95% CI)	*p* Value Regular Socks vs. Biomechanics Socks Mean (Newtons) ± SD (95% CI)	*p* Value Barefoot vs. Biomechanics Socks Mean (Newtons) ± SD (95% CI)
Newtons (N)	20.21 ± 2.72	19.62 ± 5.18	13.33 ± 3.54	0.329	<0.001 **	<0.001 **
	(8.46–22.5)	(9.38–29.9)	(6.98–22.43)			

Abbreviations: **SD** = Standard Deviation; **CI** = Confidence Interval; ***p*** **value** = level of significance; *p* < 0.05 was considered statistically significant, and *p* < 0.001 ** was considered strongly statistically significant.

## Data Availability

The datasets used and analyzed during the current study are available from the corresponding author upon reasonable request.

## Abbreviations

MTPJ	metatarsophalagic joint
FPI	Foot Posture Index
SD	Standard Deviation
ICC	Intraclass Correlation Coefficient
SEM	Standard Error of Measurement
MDC	Minimal Detectable Change
CI	Confidence Interval
